# Latham on Diseases of the Heart

**Published:** 1847-04

**Authors:** 


ART. VI.—*Latham on diseases of the Heart.* Philadelphia: Ed. Barrington and Geo. D. Haswell, 1847. 8 vo. pp. 365.

This republication is the conclusion of the "Select Medical Library," edited by John Bell, M. D., which, in connection with the "Bulletin of Medical Science," has been continued for several years past. Always judicious in his selections, as the popularity of the "Library" has abundantly shown, Dr. B. in the present instance, as we think, has chosen a work which will be particularly acceptable to the Profession. Dr. Latham enjoys an enviable reputation as a careful observer, a sound thinker, and a successful teacher of clinical medicine. He has long devoted especial attention to diseases of the heart, and his labors have contributed in no inconsiderable degree to the great precision which late investigations have given to the diagnosis of the different pathological lesions of that organ.

To the Student, or Practitioner, desirous to avail himself of all the modern revelations in this interesting and important branch of practical medicine, the work of Dr. Hope is preeminently to be recommended. It is a truly great, incomparable work! Yet there is doubtless justice in the remarks referring to it contained in the following portion of Dr. Latham's preface, which we quote as exhibiting the design and scope of the work by Dr. L.:

"Of the Lectures now published, some are literally, and some are only in substance, those which were given at St. Bartholomew's; and others have been added to them.

"Their subject is, diseases of the heart. For after all that has been written upon it, something, I have thought, is still wanting to bring it within the easy reach of the medical student; and this I have endeavored to supply.

"The treatise of Dr. Hope is very comprehensive. It embraces all that concerns the heart, its physiology, its pathology, and the treatment of its diseases. But the very abundance of its matter has made it a *hard* book to the student, and its style, which is too often controversial, and even disputatious, repels many readers, and has been in some measure a hindrance to its usefulness. Yet it is a great work, and must always hold a high place in the medical literature of this country. Such information as I have to impart, has no aim of superseding either this or any other valuable work upon the same subject, but will rather (I trust) render the student more desirous of consulting it, and more apt to consult it profitably.

"Mine is a limited purpose. It is to regard the diseases of the heart only in one point of view, *i. e.* as they appear in the living man.—But this one point of view includes the several of their clinical history, and their medical treatment. These are what I seek especially to illustrate, while I presume an acquaintance with other parts of the subject, and shall only allude to them incidentally as I go along."

We are tempted to make farther quotations from the preface by the soundness of the views expressed, that our readers may form some idea of the Author's style of composition. He thus comments on the danger of *teaching too much*. The principle to which he refers is an important one, in any and every branch of education. True knowledge is to be *acquired*, not *communicated*; and the office of the teacher should be to aid the mind of the pupil in his own efforts of acquisition. We often hear some men characterized as self-educated; but all well educated men, in whatever department of knowledge, but more especially in practical knowledge, are, and of necessity must be, self-educated—but to the quotation:

"After I have described the auscultatory signs, and endeavoured to show what they are, both in themselves and in relation to other symptoms, and what is their value as guides both to diagnosis and treatment, I may perhaps seem unreasonable to cut short the further description of the heart's diseases.

"But as I lectured, so now I write, for one class of students especially. As my hearers were, so now I presume my readers will be, chiefly those who are seeking information at the bedside. To such there is no greater impediment of knowledge than over-teaching.— The teaching which they most require is suggestive. They have the realities themselves to learn from, the original *book* to read, upon which all sound instruction is but a commentary. Therefore the commentator should only interpose when and where he is needed, and not after the manner of certain critics, who most *help* us with their annotations where the sense of the author is clear beyond dispute.

"A country pastor made one of his flock a present of Bunyan's "Pilgrim's Progress;" and, anxious that he should both read it and profit by it, took care that the copy which he gave him should be one well furnished with notes. Meeting the man some time afterwards, he asked him how he liked the book, and whether he was sure that he understood it; and received for an answer, that he both liked it and understood it all well enough, *except the explanations.*

"So with students who have free access to the wards of a great hospital, we should not be too ready in describing and commenting upon the ordinary phenomena of diseases which are constantly before their eyes, lest, perchance, they should tell us 'that they understand all well enough, except the explanations.'"

Upon the evils of too exclusive attention to specialties he makes the following remarks, which are pertinent:

"The study of our times has been chiefly to specialise and to localise disease, and it has had very useful results. But it has had a tendency to narrow our views, and to cripple our practice, by setting up as many several pathologies within the body as there are several organs. Yet no sooner do the diseases of separate parts come to be treated, than they begin to claim their place in a common pathology. We cannot reach them, and apply our remedies directly to them, in the isolated spots wherein we find them; but if they are to be reached, and treated at all, it must be through the vascular system, or through the nervous system, or through the digestive aad assimilative system. For these are the common agents of life and increase, both healthy and unhealthy, and the common channels both of food and of medicine."

The following concluding portion of the preface relates to the relative importance of therapeutics. The primal purpose of medical science seems to be lost sight of in the superior scientific attractions of pathology and diagnosis. The enthusiastic votary of medicine is apt to forget that his aim should be not alone to become deeply versed in those two essential branches, but to make his knowledge of them subservient to the art of healing:

"All things should have a consideration bestowed upon them in proportion to their importance: the question is, whether the treatment of diseases has, upon the whole, had as much as is due to it.

"During the last quarter of a century, physicians have laboured very hard, and, upon the whole, very profitably. But their labour has been bestowed in unequal degrees, and consequently with unequal success, upon the objects which concern them. Pathology and diagnosis have had much more of their regard than treatment. Thus our knowledge of disease in its essence has been greatly enlarged, and our skill of detecting its present existence and seat in the living body, has been made more exact and sure, while our ability of influencing its progress and events by medicine has not been proportionally increased.

"But how has this happened? Is it that the mind, which is best fitted for the study of pure pathology, is naturally averse from concerning itself with practice? One would hope not; but yet it may be so. The things themselves are different, and may naturally enough please different minds. Disease is a thing of itself, and admits of being studied with little reference to other things; and this may suit one mind. The affair of treating it must necessarily suffer admixture with various accidents and circumstances of life, and cannot be conducted without constant reference to them; and this may suit another mind. But however this may be, the fact is certain, that many eminent physicians, of foreign schools especially, to whom speculatively we owe the most, practically we owe the least. Their lessons of pathology and diagnosis are copious, original, and instructive; their lessons of treatment are brief, barren, and unprofitable.

"Yet it concerns physicians, above all men, that theirs should not be a barren knowledge, but that it should claim honour of mankind from a sense of the benefit which they receive from it. Far be it from me to contend, that every piece of pathological knowledge is to be disparaged or rejected, which cannot at once be made subservient to a practical purpose. The knowledge is to be obtained at all events, and kept ready for use, whether the use come soon, or late, or never. Use, however, is the end always to be regarded, as well philosophically as morally. An age of great increase of speculative knowledge in medicine ought, surely, to be an age distinguished by some great practical benefit.

"It is much to be lamented that any eminent master of pathology, who, while he is concerned with the nature of disease, has seemed at home, and in earnest, and satisfied with his work, pleased to instruct, and gaining favour for his instruction as he goes along, should come at last to the treatment of disease as to an humbler and less worthy portion of the physician's care. For this ought not to be. Medicine, as it begins to touch upon higher interests, even the interests of life and death, should feel itself in alliance with higher motives than any which can be thought to help and quicken its pursuit as mere science. For now it claims a sort of moral respect in the handling; it calls upon the conscience as well as the intellect, for more caution to avoid error, and more fearfulness of overstepping the truth.

The treatment of diseases, rightly considered, is, in fact, a part of their pathology. What they need, and what they can bear, the kind and strength of the remedy, and the changes which follow its application, are among the surest tests of their nature and tendency."

By the foregoing extracts our readers will perceive that Dr. L. is a plain, sensible writer, whose paramount object in assuming the task and responsibility of authorship is to impart useful, practical information—to do good. The work consists of lectures, and the conversational style of this species of composition is preserved.—Such works will always be most popular and useful. We could say much respecting the interest and importance of diseases of the heart when studied with the light of physical exploration, after the clear and simple principles laid down by Hope and others. We fear that here as in some other branches of our science, numbers are repelled by exaggerated and imaginary difficulties in the pursuit. On this subject we may have something to say hereafter.—In the mean time we commend most cordially Dr. Latham's Lectures to those who may deem our commendation worthy consideration.

